# Metformin-letrozole in comparison with Metformin-clomiphene citrate in clomiphene-resistance PCOS patients undergoing IUI

**Published:** 2011

**Authors:** Robab Davar, Mojgan Javedani, Mohammad Hossein Fallahzadeh

**Affiliations:** 1 Research and Clinical Center for Infertility, Shahid Sadoughi University of Medical Sciences, Yazd, Iran.; 2 Department of Biostatistics and Epidemiology, Shahid Sadoughi University of Medical Sciences, Yazd, Iran.

**Keywords:** *Letrozole*, *Clomiphenecitrate*, *Ovarianstimulation*, *PCO*, *IUI*, *Metformin*.

## Abstract

**Background:** Polycystic ovary syndrome (PCOS) is associated with approximately 75% of women who suffer from infertility due to anovulation. Additionally, around 20– 25% of anovulatory women with PCOS do not respond at all to clomiphene citrate and are considered to be “clomiphene– resistant”. Aromatase inhibitors have been suggested as an alternative treatment to clomiphene as the discrepancy between ovulation and pregnancy rates with clomiphene citrate has been attributed to its anti-estrogenic action and estrogen receptor depletion.

**Objective:** The aim of this study is to compare results of Metformin-letrozole with Metformin-clomiphene citrate in clomiphene resistance PCOS patients undergoing IUI.

**Materials and Methods:** In this single blind randomized trial, ovarian cycles were studied in 100 clomiphene- resistant patients with PCOS. The inclusion criteria were patients who received 150mg clomiphene citrate daily for 3 cycles and failed to become pregnant. The patients were matched for their age, body mass index (BMI), and infertility period. They were randomly allocated to a metformin-letrozole group (n=50) and a metformin-clomiphene citrate group (n=50). Chemical and clinical pregnancies were assessed after IUI. Abortion rates were determined in both groups.

**Results:** Regarding pregnancy rate, there was no significant difference between the two groups. One miscarriage (2%) occurred in the metformin-clomiphene citrate group, whereas none was seen in the metformin-letrozole group.

**Conclusion: **There is no significant difference in pregnancy rate between clomiphene citrate and letrozole groups although it has been 2% in the former and 5% in the latter.

## Introduction

Polycystic ovary syndrome (PCOS) is associated with approximately 75% of women who suffer from infertility due to anovulation (1, 2). The introduction of small amounts of FSH indirectly with clomiphene citrate into the circulation is capable of inducing ovulation and pregnancy in large number of anovulatory women with PCOS (3). Ovulation is restored in approximately 80% but results in pregnancy in only about 35–40% of patients who are given clomiphene (3-5). 

 Additionally, around 20–25% of anovulatory women with PCOS do not respond at all to clomiphene citrate and are considered to be 'clomiphene resistant' (6, 7). Patients who do not respond to clomiphene are likely to be more obese, insulin- resistant, and hyperandrogenic than those who do respond (7). 

 Numerous studies in women who were treated with clomiphene citrate and in various model systems have reported that it causes thick and scanty cervical mucus, thinning and inappropriate growth and maturation of endometrium. (8-13). It is generally believed that these effects are most apparent at higher doses or after longer durations of treatment with clomiphene citrate (9-11). 

 The endometrium is believed to be one of the most important targets of the antiestrogenic effect of clomiphene citrate and may explain a large part of the lower pregnancy rate and the possible higher miscarriage rate with clomiphene citrate. A reduction in endometrial thickness below the level thought to be needed to sustain implantation was found in up to 30% of women receiving clomiphene citrate (10). This observation has been confirmed by other studies such as Nelson *et al* and Li *et al* (12, 13).

Aromatase inhibitors have been suggested as an alternative treatment to clomiphene as the discrepancy between ovulation and pregnancy rates with clomiphene citrate has been attributed to its anti-estrogenic action and estrogen receptor depletion. 

The aromatase inhibitors do not possess the adverse anti-estrogenic effects of clomiphene but, by suppressing estrogen production, mimic the central reduction of negative feedback through which clomiphene works. Letrozole, the most prevalently used anti aromatase for this indication, has been shown to be effective, in early trials, in inducing ovulation and pregnancy in women with anovulatory PCOS and inadequate clomiphene response (14) and improving ovarian response to FSH in poor responders (15). Evidence from larger trials is still awaited, but some encouragement may be taken from the solidity of the working hypothesis and the success of the preliminary results. The aim of this study is to compare results of metformin-letrozole with metformin-clomiphene citrate in clomiphene resistance PCO patients undergoing IUI.

## Material and methods

 In this single blind randomized trial, 148 ovarian cycles were studied in 100 clomiphene- resistance patients with PCOS who were chosen among 250 PCOS patients attending the Research and Clinical Center for Infertility, Shahid Sadoughi University of Medical Sciences, Yazd, Iran during the years 2007-2008.

 The Study was approved by Ethical Board of Shahid Sadoughi University of Medical Sciences, Yazd. Also it was fully supported and funded by Shahid Sadoughi University of Medical Sciences, Yazd, Iran.

**Study samples**

 In this study, 50 cases were needed in each group so as to gain a significant difference of 22% in pregnancy rate at a significant level of 5% and a power of 80%. Randomization was conducted on the basis of random numbers table right after metformin administration. The diagnosis of PCOS were based on oligo and /or anovulation, high androgen level and more than 10 antral follicles which are in accordance with the revised 2003 Rotterdom criteria of PCOS. Thyroid, liver, kidneys function, and prolactin level were checked for normal values. Samples without any of them were excluded from the study. Data were collected from the samples via questionnaires along with conversation and physical examination done by a medical researcher. Hysterosalpingography and sperm analysis of our samples were normal. 

Inclusion criteria were patients who received 150 mg clomiphene citrate daily for 3 cycles and failed to become pregnant, that considered as clomiphene failure. In this study, however, the selected cases took the drug in just one treatment cycle. 

We excluded patients with liver and kidney dysfunction, cardiovascular disease, diabetics, and those who use metformin or drugs affecting insulin secretion and clomiphene citrate in recent 2 cycles.

**Study protocol**

The patients were randomly allocated to metformin–letrozole group (n=50) and metformin-clomiphene citrate group (n = 50). 

All the patients of both groups received 1500 mg/day metformin for 6–8 weeks. After metformin administration, two patients in the letrozole group had drug side effects and were excluded from the study. If metformin alone results in pregnancy, the patients were excluded. If pregnancy did not occur, the patients in the metformin– clomiphene group received clomiphene citrate100mg daily from cycle day 3-7, and metformin–letrozole group were given letrozole 5mg daily from cycle day 3-7. The drug administration continued in three cycles for all the included patients. Monitoring of follicular development was done by transvaginal sonography. When there was at least one follicle≥18mm HCG 10000 unit was administered. Thickness of endometrium, number of follicles >18mm, estradiol level, and chemical and clinical pregnancy rate were determined.

Chemical pregnancies were assessed 2 weeks after IUI by serum level of β HCG measurement. Existence of at least one gestational sac in ultrasonography was confirmed as a clinical pregnancy. Ongoing pregnancy was defined as the observation of at least one fetus with heart activity in sonography after the first trimester of pregnancy. Abortion rates were determined in both groups. 

**Statistical analysis**

 The sample was divided into two groups using the random allocation software. SPSS version 12.0 software was used for the statistical analysis. *T*-test, chi-square and Fisher exact tests were used when appropriate. P-values less than 0.05 were considered as statistically significant.

## Results

On the whole, 148 ovarian cycles were studied in 98 patients (70 cycles in 48 patients in the metformin–letrozole group and 78 cycles in 50 patients in the metformin–clomiphene citrate group).

Ninety eight PCO patients who were allocated to the two groups were included in the study. All the patients had at least 2 out of 3 Rotterdom criteria. No significant difference was seen between the two groups according to age, BMI, and infertility duration. 

Estradiol level on HCG day was significantly higher in the metformin– clomiphene group as compared with those in the metformin–letrozole group (175.01±168.93 vs. 70.24±138.32 pg /ml; p<0.05) (Table I). Mean endometrial thickness was also significantly lower in the metformin– clomiphene group than those in the metformin–letrozole group (0.9±9.3 cm vs. 1.03±10.2 cm; p< 0.05) (Table I). 

One miscarriage (2%) occurred in the metformin–clomiphene citrate group, whereas none were seen in the metformin– letrozole group. The clinical pregnancy rate was not significantly different between the two groups [4 patients (8.3%) in metformin–letrozole group as compared to 1 patient (2%) in metformin– clomiphene group; p > 0.05)] (Table II). 

The pregnancy rate per cycle was 1% (1of 78 cycles) in the clomiphene-citrate group but 5% (4 of 70 cycles) in the letrozole group, which was not statistically significant.

**Table I:** Comparison of baseline parameters and different variables in the metformin-clomiphene citrate group and the metformin–letrozole group based on mean ± SD

**Variable**	**Metformin-clomiphene group**	**Metformin-letrozole group**	**p-value** *
Number	50	48	-
Age (years)	29.55 ± 3.47	28.54 ± 3.13	NS*
BMI	29.21 ± 2.92	28.98 ± 3.83	NS*
Endometrial thickness on day of HCG administration (mm)*	9.3 ± 0.9	10.2 ± 1.03	<0.05
Duration of infertility (years)	3.81	3.76	NS*
Mean E2 (pg/ml) on day of HCG administration	168.93 ± 175.01	138.32 ± 70.24	<0.05
Number of follicles > 18 mm in diameter	1.85 ± 0.27	1.80 ± 0.32	NS*

* P-value: NS=not significant.

**Table II:** Comparison of pregnancy and miscarriage rate in two groups based on mean ± SD

**Variable**	**Metformin-clomiphene group**	**Metformin-letrozole group**	**p-value** *	**Relative risk** **0.95 CI (0.5-34.5)**	**Risk reduction**
Regular menses after metformin	36 (75%)	36 (72 %)	0.736	-	-
Adverse effect	0(0%)	2 (4%)	0.495	-	-
Chemical pregnancy rate	4%	8%	0.677	-	-
Clinical pregnancy rate	2%	8.3%	0.362	4	6.25
Intension to treat analysis	1 (2%)	4 (8%)	0.362	4	6.25

* P-value: No difference was found between the two groups for the above parameters (by exact t-test as appropriate).

## Discussion

This study showed that in PCOS with clomiphene-failure, combination of metformin–letrozole results in higher pregnancy rates and less abortion than metformin-clomiphene. No significant relationship was observed between age, BMI, and duration of infertility in the clomiphene citrate and the letrozole groups. Endometrial thickness in clomiphene citrate group was less than letrozole group. Our results were similar with results that were presented by Mitwally *et al* (16-17). But in the study that was done by Al-Fozan *et al* a significant relationship was not reported between these two groups. Fisher *et al* in their study believed that “the cause of endometrial thickening in patients receiving letrozole is because of improved vascularization as compared with clomiphene citrate” (18). Some studies showed that clomiphene citrate can cause inadequate endometrial thickness in 15–50% of patients (19). 

Cervical mucus became thick and scant and decrease of endometrial thickness occur (14). These side effects may be due to the anti-estrogenic effect of clomiphene citrate and also longer half-life, that leads to lowering number of estrogen receptors and decreasing endometrial thickness (14). 

One miscarriage was happened in the metformin–clomiphene citrate group whereas none were seen in the metformin-letrozole group. The clinical pregnancy rate between the two groups did not show significant difference. In the study by Mitwally and Casper the effect of letrozole administration in 10 women with PCOS was assessed. They found that pregnancy occurred in 20% of women (14). Sammour *et al* in their study on efficacy of letrozole and clomiphen citrate in 49 women with idiopathic infertility showed that pregnancy rate was higher in letrozole group than clomiphene citrate group (16.7 vs. 5.6%) (20). According to the results of those studies; pregnancy rate is higher in patients receiving letrozole than clomiphene citrate group but is lower when compared with our study. Moll *et al* showed that in clomiphene citrate-resistant women, the combination of clomiphene plus metformin was the preferred treatment. Some studies reported that rate of miscarriage was higher than expected in clomiphene group. In this study, there was one case of abortion in clomiphene group. This increase may be due to low estrogen level in the cervical and endometrial mucosa. There is some evidence that clomiphene citrate has deleterious effect on the the initial stages of development in mouse and rabbit but has not been proved (21). We hypothesize that clomiphene may have direct adverse effects on oocytes. Some studies stated that low pregnancy rate in patients receive clomiphene is due to inadequate uterine blood flow during the early luteal phase (21). Women with oligomenorrhea and PCOS had some ovulatory disorder because of insulin resistance and its related factors (22). Serum insulin level was main role in PCOS pathogenesis. “Metformin can increase tissue sensitivity to insulin as well as decrease plasma insulin level and hepatic glucose production. In PCOS, metformin can decrease the level of LH and ovarian androgen level as well as correct hyperinsulinemia” (23). Some studies showed the effect of metformin on the function of ovaries (22). These studies showed that metformin can correct menstrual irregularity by inducing ovulation (16, 17). According to Nestler study, ovarian response to clomiphen was higher in obese women with PCOS (24). 

Aromatase enzyme converts androstenedione to estrone and then to E2 in peripheral tissues. Therefore aromatase inhibitors prevent estrogen production in these tissues. According to noted mechanism, some of selective aromatase inhibitors such as letrozole are used to induce ovulation especially in infertile women with PCOS (21). They may have direct effect on the ovaries and increase follicular sensitivity to FSH. The level of ovarian aromatase is low in these patients. Multiple small ovarian follicles is due to high androgen level. In addition, androgens increase FSH receptors, and therefore increase FSH sensitivity. Aromatase inhibitors cause growth of one or more ovarian follicles by increasing FSH or deceasing estrogen production (25, 26). 

Our Results show that letrozole can be an alternative to clomiphene citrate, especially in clomiphene failures, or it can be used in anovulatory patients at first. It seems that letrozole is a safe, reliable and inexpensive drug with therapeutic value (16, 17). Serum clearance rate of letrozole is greater than clomiphene citrate (50hour for letrozole versus 4 weeks for clomiphene citrate) and because letrozole does not decrease estrogen receptors, it has no deleterious effects on the endometrium in comparison with clomiphene citrate, hence lead to higher pregnancy rate (4, 12). Because we didn’t have one optimum dosage for therapeutic effects of letrozole, further studies are necessary for achieving that (16,17).

Moreover IUI had no favorable success and because IUI was not considered as a therapeutic modality in recent references (27), it is better to do IVF or ICSI in patients who do not respond to medical therapy.
